# Does the Rural Environment Influence Symptomatology and Optimize the Effectiveness of Disease Acceptance? A Study Among Women With Fibromyalgia

**DOI:** 10.3389/fpsyg.2021.658974

**Published:** 2021-04-29

**Authors:** Patricia Catalá, Sheila Blanco, Soledad Perez-Calvo, Octavio Luque-Reca, Dolores Bedmar, Cecilia Peñacoba

**Affiliations:** ^1^Department of Psychology, Rey Juan Carlos University, Madrid, Spain; ^2^Pain Unit, Hospital Universitario de Fuenlabrada, Madrid, Spain

**Keywords:** rural/urban health, fibromyalgia symptoms, women, pain, disease acceptance

## Abstract

The present study aims to explore whether the symptoms associated with fibromyalgia are contextually influenced by the area of residence (rural/urban). Furthermore, it is analyzed whether the effect of the acceptance of the disease on the emotional, cognitive and physical symptoms is moderated by the patients’ place of residence. Using a cross-sectional design, a total of 234 women with fibromyalgia (mean age = 56.91 years; SD = 8.94) were surveyed, of which 55.13% resided in rural areas and 44.87% in urban areas. Self-reported questionnaires were used to assess pain severity, anxiety and depression, functional limitation, physical and mental fatigue and acceptance of the disease. The results show significant differences in acceptance (*p* = 0.040), pain (*p* < 0.001), and physical and mental fatigue (*p* = 0.003 and *p* = 0.004, respectively) between patients from rural and urban areas. The rural area patients presented higher levels of acceptance and pain and lesser levels of physical and mental fatigue compared to the urban area. The moderation analysis add that, only in patients from the rural area, the variables of physical symptoms (pain, functional limitation, and physical fatigue) were significantly and negatively associated with acceptance. This study addresses for the first time the role of the place of residence in suffering from fibromyalgia, suggesting that the rural or urban environment plays a relevant role in the severity and/or management of symptoms in fibromyalgia women. Limitations and practical implications are also discussed.

## Introduction

Fibromyalgia (FM) is considered a chronic disease characterized not only by chronic widespread pain but also by other symptoms such as fatigue, sleep problems, anxiety, or depression. Fibromyalgia causes a negative impact on the quality of life of patients (Campos and Vázquez, 2012; Arnold et al., 2016) and has been linked to substantial impairments in both physical and mental health and functionality (Wolfe et al., 2014; Walitt et al., 2015).

From the recognition of FM as a disease to the present, the scientific community has tried to identify which biopsychosocial factors are involved in its appearance, maintenance and severity (Fitzcharles et al., 2014; Häuser et al., 2015). It is known that, in FM patients, well-being and adaptation to the disease are related to affective, cognitive and behavioral variables (Thompson and McCracken, 2011). For example, fear related to pain, impotence, or passive coping strategies such as avoidance, increase pain levels and decreases functionality (Mayangsari et al., 2019; Higuchi, 2020). Instead, active coping strategies (e.g., mindfulness), self-efficacy, willingness to change or acceptance of illness have been shown to reduce psychological distress, pain, or functional limitation (Dorado et al., 2018; Van Liew et al., 2019). Specifically, acceptance, understood from the model of psychological flexibility integrated into acceptance and commitment therapy (ACT) (Hayes et al., 2016), refers to the ability to learn to live with discomfort without the need to reduce, avoid or try to change it (McCracken and Velleman, 2010). Studies with mixed samples of patients with chronic pain show that higher levels of acceptance are associated with better daily functioning and less disability and symptoms (Kratz et al., 2007). In patients with fibromyalgia specifically, the previous literature is consistent with the positive role of acceptance in the suffering (Tangen et al., 2020). In this vein, it seems that accepting the disease reduces the levels of anxiety, depression and pain and improves the functioning of these women (Rodero et al., 2011; Yu et al., 2017; Lami et al., 2018; Trainor et al., 2019).

The literature also suggests that the relationship established between psychological factors and health outcomes is not always linear, but depends, to a large extent, on the context (Suso-Ribera et al., 2017). As suggested by the environmental competence model (Lawton, 1986; Moore et al., 2003), the environment-person relationship is determined by the relationship between the level of personal competence and environmental demand. While the level of personal competence depends on health, sensory capacity, motor performance, and cognitive abilities, environmental demand is determined by actual and perceived physical characteristics (Lawton, 1977; Rousseau et al., 2002). From this model, it is hypothesized that the lower the level of competence, the greater the influence of contextual factors on well-being. Therefore, the physical environment (i.e., residence area) would have a special influence on people with a worse state of physical or mental health. Specifically, Lawton’s Competence and Environmental model (Lawton, 1986) has been used as a conceptual framework especially in relocation and gerontology (Perry et al., 2014). As an example, this model has proven useful to explain the relationship between person-environment fit and apathy (Jao et al., 2020) and well-being (Calkins, 2018) in long-term care residents with dementia, and for the ability to comply with a medication regimen in elderly persons (LeRoux and Fisher, 2006). Recent literature confirms the differential role of the environment in patients with multiple chronic conditions. In general terms, it seems that rural patients have a worse physical and mental health status compared to urban participants (Wang et al., 2015; Cheng et al., 2020). To our knowledge, the Lawton’s Competence and Environmental model (Lawton, 1986) has not been used in patients with fibromyalgia.

Given the increasing prevalence of FM, both in rural (between 0.1 and 5.2%) and urban (between 0.7 and 11.4%) populations (Marques et al., 2017), the first aim of the present study has been to explore whether the symptoms associated with this syndrome (i.e., pain, anxiety, depression, functional limitations, and physical and mental fatigue) are contextually influenced by the area of residence (rural/urban). Furthermore, as a secondary objective, it is analyzed whether the effect of the acceptance of the disease on emotional, cognitive, and physical symptoms is moderated by the residence area of these patients.

## Materials and Methods

### Study Design

The study design was a cross-sectional cohort. The Ethics Committee of the Rey Juan Carlos University approved the study protocol and evaluation procedures (Reference PI17/00858; number 160520165916). In this study, the Strengthening of the Notification of Observational Studies in Epidemiology (STROBE) guidelines for cross-sectional studies was followed and applied (Von Elm et al., 2008).

### Participants

Two hundred thirty-four women with FM were recruited with a mean age of 56.91 years (SD = 8.94). Of these, 129 participants (55.13%) reside in rural areas and 105 (44.87%) reside in urban areas. Six percent of the women had completed higher education studies, 29% completed secondary studies and 65% primary studies. Fifty-three percent of the women were married or in a stable relationship, 11% were single, and 36% of them were divorced or widowed. The vast majority of the participants were housewives (78%). Participants had a diagnosis of FM for an average of 12.14 years (SD = 8.45; range 1 to 46 years). The mean intensity of perceived pain was 7.28 (SD = 1.74, range 0–10).

### Eligibility Criteria

Only female adults were included in this study (for homogeneity purposes because almost all FM patients are females). The eligibility criteria to participate in the present study included having a diagnosis of FM according to the American College of Rheumatology (ACR) criteria (Wolfe et al., 1990, 2010), being over 18 years of age, and providing a written consent to participate in the investigation. In addition, as exclusion criteria, not having the physical and mental ability to provide informed consent and to complete the surveys was included.

### Procedure

A convenience sample was selected by contacting several patient associations from different Spanish regions during the years 2018 and 2019. In total, 268 participants agreed to participate in the study and met our initial inclusion criteria. Finally, effective responses were obtained from 234 patients (25 patients did not attend the scheduled evaluation appointment, 6 questionnaires were left blank, and 3 questionnaires contained a large amount of missing data that could not be retrieved because the data could no longer be reached participants). The initial sample size was calculated based on recommendations for similar studies (Westland, 2010). The 234 participants were classified according to the place of residence (rural area/urban area), based on the Publication of Urban Areas in Spain 2019, published by the Ministry of Transport, Mobility and Urban Agenda, DG of Housing and Floor (Ministerio de Transportes, Movilidad y AgendaUrbana and Direccion General de Vivienda y Suelo, 2020). Once the participants gave their informed consent to participate in the study, they were given a questionnaire booklet that took approximately 30 min to complete. The questionnaires were completed in the associations of the different regions evaluated, they were carried out individually and with the supervision of a professional psychologist (in groups of between eight and ten patients).

### Measures

#### Pain

To assess pain intensity, the mean score of the four pain intensity items from the Brief Pain Inventory (BPI) was used (Cleeland and Ryan, 1994): maximum, minimum, and overall pain intensity during the last 7 days and pain intensity at the current time. Each rating is evaluated using an 11-point numerical scale (0 = “no pain” and 10 = “the worst pain you can imagine”). This procedure to measure pain severity has been widely used in the pain literature (Jensen et al., 1996). In this study, the internal consistency of this scale was high (0.86).

#### Anxious and Depressive Symptoms

The Spanish version of the Hospital Anxiety and Depression Scale (HADS) was used to measure the presence and severity of symptoms of anxiety and depression (Herrero et al., 2003). The HADS is a 14-item questionnaire that comprises two subscales: the HADS-A (7 items) that measures anxiety and the HADS-D (7 items) that measures depression. Participants were asked to rate the degree to which they experienced various emotions in the past week. The scale is composed of items such as “I feel tense or nervous” for anxiety or “I have lost interest in my appearance” for depression. All items were scored on a 4-point Likert scale (0–3). Scale scores were calculated by summing the scores on the individual items of a subscale. Scale score range from 0 to 21 with higher scores indicating more symptoms. This questionnaire has shown good psychometry properties (Herrero et al., 2003). Cronbach’s α coefficients for HADS-A and HADS-D in this study were 0.87 and 0.85, respectively.

#### Functional Limitation

The dimension of “physical function or functional limitation” of the Spanish adaptation of the Revised Fibromyalgia Impact Questionnaire (FIQ-R) was used for this study (Salgueiro et al., 2013). The FIQ-R questionnaire consists of 21 items with a Likert response format of 11 points (from 0 to 10) that assesses three associated domains: physical function, general impact and symptoms. The physical function (used in this study) is the sum of the first 9 items divided by 3, and can take a value between 0 and 30. It evaluates the degree to which they were experienced difficulties when carrying out a series of basic physical activities of daily life (for example, “shopping” or “climbing stairs”). Higher scores indicate less functionality. Cronbach’s alpha in the present study was 0.88. The sub-scale has obtained good reliability and validity indices in the past (Ciapetti et al., 2013).

#### Physical and Mental Fatigue

For this study, the dimensions of physical and mental fatigue were selected from the Spanish version of the Multidimensional Fatigue Inventory (MFI) (Munguía-Izquierdo et al., 2012). This questionnaire is a 20-item assessment tool with a 5-point Likert (1–5) response scale. It evaluates five domains of fatigue: general fatigue, physical fatigue, mental fatigue, reduced motivation and reduced activity. Each dimension consists of four elements and the score for each one of them ranges from 4 to 20 points. Higher scores indicate a high degree of fatigue symptoms. The physical fatigue subscale contains items such as “Physically, I feel like I can only do a little bit” and the mental fatigue subscale contains items such as “I can concentrate well”. The selection of these two dimensions was motivated by their conceptualization of fatigue as a physical and mental symptom, according to the previous literature on the relationship between both physical and mental fatigue and acceptance (Brooks et al., 2011). In the present study, the internal consistency of physical fatigue was 0.68 and of mental fatigue was 0.73.

#### Acceptance

For this study, the subscale of “commitment to activity” of the Spanish version of the Chronic Pain Acceptance Questionnaire (CPAQ) was selected (McCracken et al., 2004). The CPAQ is a 20-item self-report questionnaire that assesses the acceptance of chronic pain. The questionnaire is made up of two subscales, the pain disposition subscale (11 items) and the activity commitment subscale (9 items). The last, used for this study, contains items such as “Although things have changed, I am living a normal life despite my chronic pain.” All items are rated on a scale from 0 (never true) to 6 (always true). This measure provides total scores ranging from 0 to 54; higher results mean high pain acceptance. Cronbach’s alpha for this study of the commitment to activity subscale was 0.76. The validity and reliability of this instrument has been demonstrated in the past (Rodero et al., 2010).

#### Sociodemographic and Clinical Data

An *ad hoc* questionnaire was used to evaluate age, place of residence, educational level, employment status, and marital status. Regarding clinical variables, duration of FM was recorded.

### Statistical Analysis

For data analysis, SPSS version 22.0 statistical program was used (IBM Corp, 2013). The comparisons between the rural area and urban area groups in the sociodemographic variables were made using the *t*-test and the Chi-square test for continuous and categorical variables, respectively. For the analysis of differences between the rural and urban groups in the symptoms of the disease controlling for age, a one-way multivariate analysis of covariance (MANCOVA) was conducted. Effect size differences were assessed using η*_*p*_^2^* (η*_*p*_^2^* = 0.01 small effect, η*_*p*_^2^* = 0.06 medium effect, and η*_*p*_^2^* > 0.13 large effect) (Cohen, 1988).

Next, a series of multivariate regressions were computed with model 1 of the PROCESS macro (Hayes, 2017). Bootstrap-based bias-corrected confidence intervals (95%) were generated for indirect effects using 5000 iterations of bootstrap. In each regression, a combination of the independent variable (i.e., acceptance), the moderator (i.e., rural and urban area), and their interaction, controlling for the sociodemographic variables, were entered to predict the study outcome (i.e., pain, anxiety, depression, physical and mental fatigue, and functional limitation). *Post hoc* analyses were then computed when a significant moderation was found. This was done to obtain the conditional effects of the independent variables on outcomes at different levels of the moderator and to graphically represent the moderation findings.

## Results

### Differences in Sociodemographic Variables Between the Rural Area and the Urban Area Group

Table 1 shows the significant differences in the sociodemographic variables between the patients from rural and urban areas. Specifically, the findings show significant differences in age (*t* = 4.20, *p* < 0.001), educational level (Chi-square *p*-value < 0.001), employment status (Chi-square *p*-value < 0.001) and marital status (Chi-square *p*-value = 0.014). Specifically, patients from rural areas are older and have a lower educational level; furthermore, they are mostly housewives and are married.

**TABLE 1:** Differences in sociodemographic variables between the rural area and the urban area group.

	**Rural areas (*n* = 129)**	**Urban areas (*n* = 105)**	***p*^*a*^**
Age, mean (SD)	59.04 (SD = 9.32)	54.19 (SD = 7.65)	< 0.001
Educational level, n (%)			<0.001
Primary education	101 (78.29)	51 (48.57)	
Secondary education	25 (19.38)	42 (40)	
University education	3 (2.33)	12 (11.43)	
Employment status, n (%)			<0.001
Working	7 (5.42)	21 (20)	
Sick leave	6 (4.65)	17 (16.19)	
Domestic work	116 (89.93)	67 (63.81)	
Takes care of work at home, n (%)			
Yes	115 (89.14)	98 (93.33)	0.146
No	14 (10.86)	7 (6.67)	
Marital status, n (%)			
Married or in a stable relationship	108 (83.72)	74 (70.48)	0.014
Single	5 (3.88)	9 (8.57)	
Divorced or widowed	16 (12.40)	22 (20.95)	

*^*a*^*p*-values of the *t*-test or the Chi-square test for quantitative and categorical variables, respectively.*

### Differences in Acceptance Between the Rural Area and the Urban Area Group

The results show significant differences in acceptance regarding the area of residence (*F* = 5.13, *p* = 0.007, η*_*p*_^2^* = 0.05), obtaining the rural area (mean = 32.66, SD = 11.23) higher levels of acceptance with respect to the urban area (mean = 29.40, SD = 12.31).

### Differences in Disease Symptoms Variables Between the Rural Area and the Urban Area Group

As shown in Table 2, rural patients obtained significantly higher scores in pain (*p* < 0.001) and lower in physical fatigue (*p* = 0.008) and mental fatigue (*p* = 0.008). Pain has the highest effect size (medium effect). No statistically significant differences were observed in the rest of the symptoms considered.

**TABLE 2:** Differences in symptoms between patients from rural and urban areas.

	**Rural areas (*n* = 129) Mean (SD)**	**Urban areas (*n* = 105) Mean (SD)**	***F***	***p***	***η_*p*_^2^***
Pain	7.48 (1.52)	6.75 (1.42)	13.76	< 0.001	0.06
Anxiety	12.38 (3.95)	12.00 (3.76)	0.56	0.455	0.01
Depression	9.07 (4.23)	9.49 (4.35)	0.53	0.467	0.01
Functional limitation	21.31 (5.79)	21.64 (5.79)	0.47	0.486	0.01
Physical fatigue	14.59 (3.46)	15.93 (3.16)	8.99	0.003	0.04
Mental fatigue	14.69 (3.08)	15.89 (3.13)	8.39	0.004	0.04

### Multivariate Linear Regression and Moderation Analysis

Table 3 shows the results of the regression analysis, including moderations, controlling for the sociodemographic variables (i.e., age, educational level, employment status, and marital status). Analysis revealed a direct (negative) effect of acceptance on anxiety (*p* = 0.008), depression (*p* = 0.018) and mental fatigue (*p* < 0.001). No direct effects of acceptance were observed on pain, functional limitation and physical fatigue (all *p*’s > 0.05). That is, once the sociodemographic variables have been controlled, acceptance is negatively related to anxiety, depression, and mental fatigue, regardless of the place of residence (rural/urban).

**TABLE 3:** Prospective prediction of fibromyalgia symptoms from acceptance, rural areas, and their interaction.

	***R*^2^**	***F***	***p***	**Beta**	***t***	***p***	**95% CI**
*DV* = *Pain*	0.09	7.80	< 0.001				
Acceptance				0.01	0.553	0.580	–0.03, 0.05
Residence area				0.72	3.67	< 0.001	0.33, 1.10
Interaction				–0.06	–2.41	0.016	–0.11, –0.01
*DV* = *Anxiety*	0.06	5.103	0.002				
Acceptance				–0.13	–2.66	0.008	–0.22, –0.03
Residence area				0.22	0.44	0.656	–0.76, 1.21
Interaction				< 0.01	0.11	0.913	–0.12, 0.13
*DV* = *Depression*	0.05	4.335	0.005				
Acceptance				–0.12	–2.37	0.018	–0.23, –0.02
Residence area				–0.59	–1.07	0.285	–1.69, 0.50
Interaction				< 0.01	0.03	0.972	–0.14, 0.14
*DV* = *functional limitation*	0.05	4.212	0.006				
Acceptance				–0.02	–0.26	0.795	–0.16, 0.12
Residence area				–0.08	–0.11	0.915	–1.54, 1.38
Interaction				–0.21	–2.18	0.029	–0.39, –0.02
*DV* = *Physical fatigue*	0.14	11.980	< 0.001				
Acceptance				–0.07	–1.66	0.096	–0.14, 0.01
Residence area				–1.39	–3.31	0.001	–2.22, –0.56
Interaction				–0.11	–2.04	0.042	–0.21, –0.01
*DV* = *Mental fatigue*	0.09	7.363	< 0.001				
Acceptance				–0.08	–2.231	0.027	–0.16, –0.01
Residence area				–1.23	–3.042	0.002	–2.02, –0.43
Interaction				–0.02	–0.395	0.692	–0.12, 0.08

*Residence area: 0 (urban area), 1 (rural area).*

Regarding the moderation analyzes, the results revealed that the residence area moderated the relationship between acceptance and pain (*p* = 0.016), acceptance and functional limitation (*p* = 0.029), as well as between acceptance and physical fatigue (*p* = 0.042). The relationship between acceptance and anxiety, depression and mental fatigue was not moderated by area of residence (all *p*’s > 0.05). That is, once the sociodemographic variables have been controlled, the relationship between acceptance and pain, functional limitation, and physical fatigue depends on the area of residence.

The evaluated models predicted a significant variance of 9% for pain, 5% for functional limitation and 14% for physical fatigue (all *p*’s < 0.01).

**FIGURE 1:**
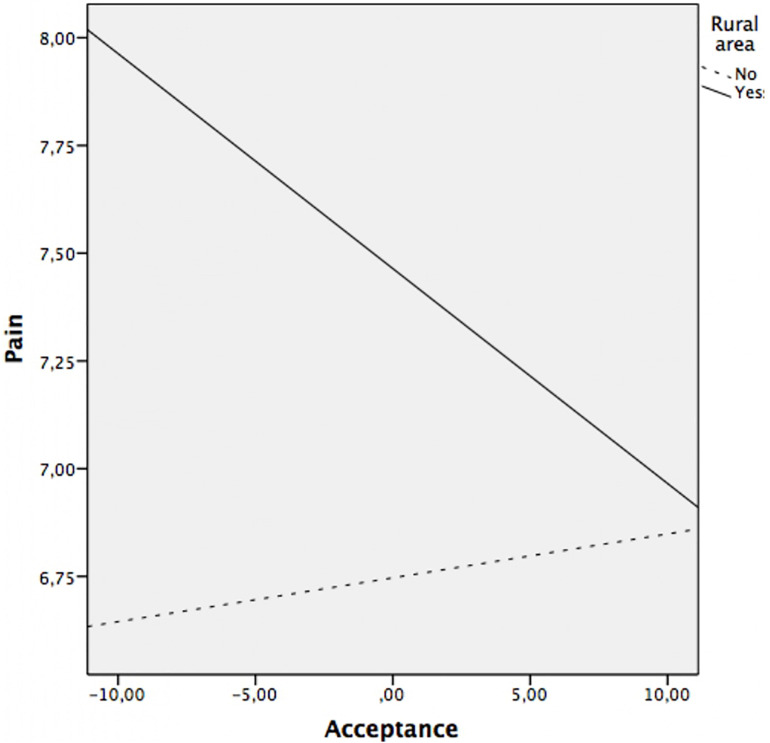
Relationship between acceptance and pain at rural area (yes/no).

**FIGURE 2:**
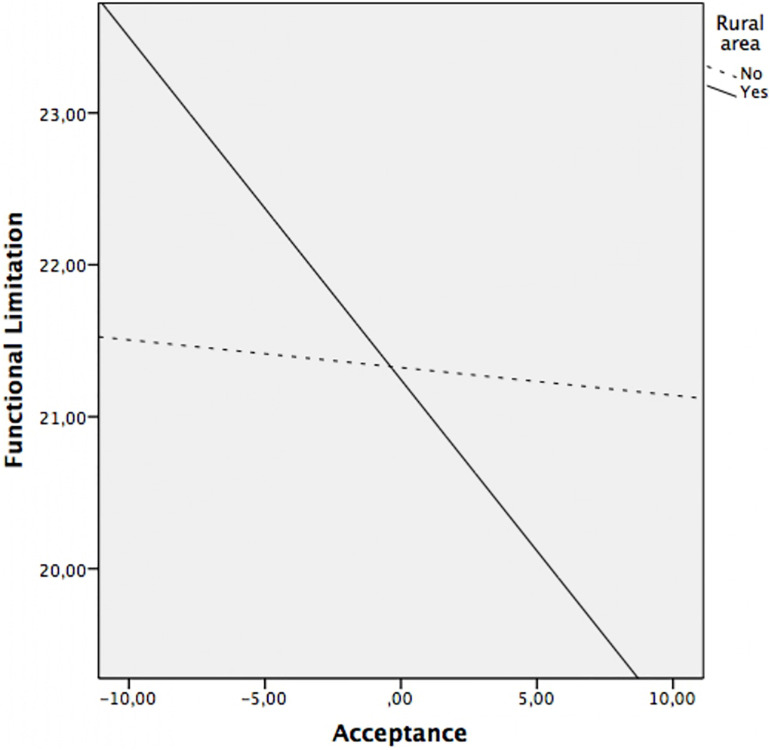
Relationship between acceptance and functional limitation at rural area (yes/no).

**FIGURE 3:**
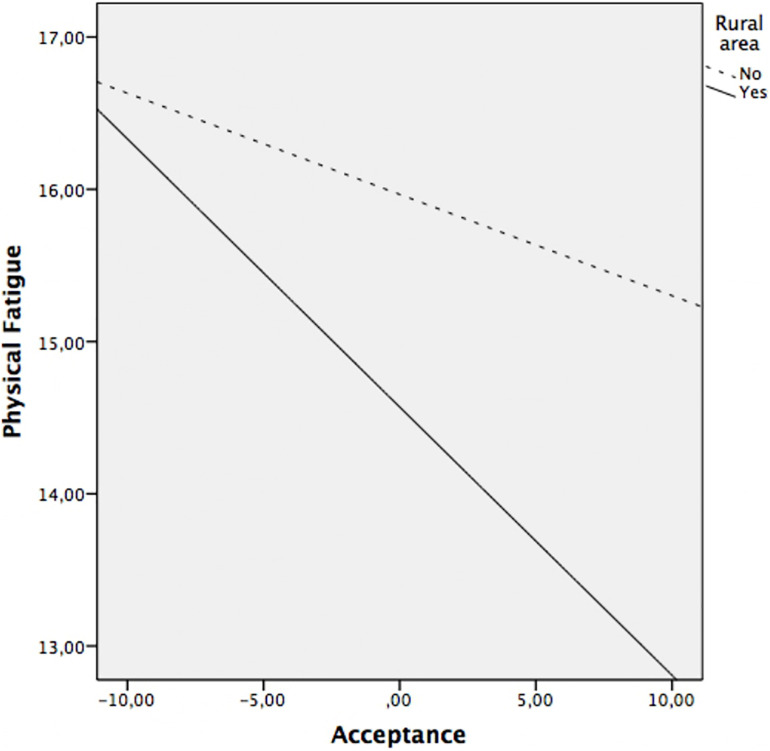
Relationship between acceptance and physical fatigue at rural area (yes/no).

*Post hoc* analysis were planned to analyze significant moderations in greater depth and are presented in Table 4 and Figure 1 for acceptance on pain, in Table 4 and Figure 2 for acceptance on functional limitation and in Table 4 and Figure 3 for the acceptance of physical fatigue. Specifically, as indicated in these tables and figures, in rural patients a significant negative relationship is observed between acceptance and pain, functional limitation, and physical fatigue. That is to say, in rural patients, the greater the acceptance, the less the pain, the functional limitation and the physical fatigue. However, in urban patients there is no association between these variables.

**TABLE 4:** Conditional effects of acceptance on pain, functional limitation and physical fatigue at residence area (urban/rural).

**DV**	**Residence area**	**Beta (acceptance)**	***t***	***p***	**95% CI**
Pain					
	Urban area	0.010	0.553	0.580	–0.03, 0.05
	Rural area	-0.049	–2.985	0.003	–0.08, –0.02
Functional limitation					
	Urban area	-0.02	–0.259	0.795	–0.18, 0.18
	Rural area	-0.22	–3.545	< 0.001	–0.35, –0.10
Physical fatigue					
	Urban area	-0.06	–1.668	0.096	–0.15, 0.01
	Rural area	-0.18	–4.880	< 0.001	–0.25, –0.11

## Discussion

The present study provides the first exploration of contextual influences (rural/urban environment) on health symptoms and disease acceptance in FM patients. Specifically, it was found that patients residing in rural settings reported significantly higher levels of pain and lower levels of physical and mental fatigue than those residing in urban areas. To our knowledge, similar studies have not been carried out in FM patients, which makes it difficult to compare results. Despite this, previous literature in the general population also indicates that women in rural areas have higher pain scores than those in urban areas (Tripp et al., 2006; Wang et al., 2015). It could be hypothesized that these differences may be due to the different treatments for chronic pain, and for fibromyalgia in particular, that patients in rural and urban areas receive. Regarding drug treatment, there seem to be no differences, neither in primary nor specialized care, between rural and urban areas. Elective drugs for fibromyalgia are psychiatric drugs that act on central pain circuits (Calandre et al., 2014). These drugs are used for the treatment of pain and for the associated emotional symptoms (anxiety and depression) that in turn contribute to the chronification of pain (Pidolle and El Hage, 2020). However, there is a growing recognition that chronic pain (Kress et al., 2015), and fibromyalgia in particular (Arnold and Clauw, 2017), must be approached from a multidisciplinary perspective that includes professionals from medicine, rehabilitation, physiotherapy, nursing, and psychology, among others (Arnold and Clauw, 2017). The therapeutic approaches that should be given priority in fibromyalgia are exercise and cognitive-behavioral therapy (treatments with the most evidence and net benefit), always respecting a multimodal approach and reserving the use of drugs for episodes of intense pain or uncontrolled symptoms (García et al., 2016). An explicit way to operationalize this multimodal approach is Pain Units or Fibromyalgia Units, which, although still scarce, are frequently located in urban settings. This less access to multidisciplinary resources of fibromyalgia patients in the rural context is in line with the current imbalance between urban and rural areas in specialized health resources (Murawski and Church, 2009; Song et al., 2018). The location of specialized units for the care of pain in urban areas probably favors that patients resident there present less pain than patients in rural areas (Hogg et al., 2012). Specifically, feeling more cared for, having better treatments or follow-ups, feeling more supported or assisted could be the main reasons why these patients present less pain. Furthermore, despite the existence of evidence in FM patients that pain severity increases with age (Jiao et al., 2014), the analysis performed precludes that these differences in pain could be due to differences in age. Fatigue, on the other hand, being a symptom that patients can control to a certain extent, is possible that it is favored by the characteristics of the rural environment. Moreover, significant differences in employability may be affecting these results. The patients in rural areas are, for the most part, housewives, so they have more time to self-manage the activities of daily living based on their state of health. Likewise, it has been verified that rural patients report greater social support (i.e., family and friends) on a day-to-day basis (Goh et al., 2016), which could make them feel less fatigued.

Interestingly, our findings did not reveal significant differences in anxiety, depression, and functional limitation between the rural/urban groups. Previous literature in clinical and non-clinical populations is not consistent with the results obtained in this regard. Specifically, studies in patients with multiple chronic conditions report that rural subjects have more symptoms of anxiety and depression (Cheng et al., 2020). In the general population, it seems that higher levels of these symptoms occur in the urban population (Blazer, 1985; Wang, 2004). However, studies in the elderly population find contradictory results. Some find higher levels of anxiety and depression in rural populations (Li et al., 2016; Cheng et al., 2020), others in urban population and others find no relationship (Chiu et al., 2005; St John et al., 2006; Salinas et al., 2010). In addition, another study in the elderly population finds that functional limitation is less likely in urban areas (Zimmer et al., 2010). These contradictions may be due to differences in the samples. However, it is important to note that the aforementioned research is based on the responses given preferably by non-clinical population in randomized surveys. The sample in this study is inherently different as it focuses exclusively on clinical subjects diagnosed with FM. This disease is characterized by its high comorbidity with other types of emotional disorders (i.e., anxiety and depression) and physical (i.e., functional limitation) (Arnold et al., 2016), which could explain the absence of differences, in the present study, in the mentioned variables. Another explanation could be related to the treatments prescribed to mitigate the effect of this symptomatology, since being very generic treatments they are prescribed by primary care or psychiatry services and these services do not differ in rural areas from urban areas (Lawson, 2017). Based on the model of environmental competencies (Lawton, 1977), which takes into account the environment-person interaction and the interpretation that the subject makes of the availability of resources, this fact could be explained. As there is no gap in the perception of resources available to treat symptoms between the health services in both areas, patients could interpret that treatment is being equal regardless of the place of residence.

Regarding acceptance, the results show significant differences with respect to the area of residence. Specifically, patients from rural areas obtain higher scores in this variable compared to those from urban areas. Previous literature on the matter in patients with FM is non-existent. Studies carried out on samples with other diseases (e.g., cancer or mental disorders) did not find significant differences on acceptance according to the place of residence (Bogusz and Humeniuk, 2017; Cipora et al., 2018). It is important to note that, in this study, the acceptance component is analyzed as a facilitator of commitment to daily activity despite pain. In this sense, it has been pointed out that finding fewer barriers to continue with the lifestyle prior to diagnosis, favors self-management of the disease (Huygens et al., 2016). It could be hypothesized that the ability to control, organize, set times, ultimately autonomy, is more favored in the rural environment than in the urban environment. In urban settings, women with fibromyalgia have to combine their work as a housewife with their work outside the home in a greater proportion than women in rural areas. This fact could have consequences on their management capacity and on the distribution of their time, affecting the performance of tasks depending on other contextual variables, such as pain, fatigue or “having a bad day” (Sanz-Baños et al., 2016). Some items of the acceptance dimension used in our study (McCracken et al., 2004) such as “I continue to do the things of daily life regardless of my level of pain” or “I do not need to control the pain to be able to lead my life well” point in this direction. In urban women with fibromyalgia, to a greater extent, work outside the home could constitute a “demanding” context with little capacity for self-management based on their symptoms.

Regression analyzes revealed that the role of place of residence (rural/urban) in the effect of acceptance on emotional, cognitive, and physical symptoms differed by symptom. It is described that regardless of the context, acceptance has a direct negative effect on variables of emotional and cognitive symptoms (anxiety, depression, and mental fatigue) but not on physical symptoms. This is possible since the concept of acceptance (derived from ACT) is aimed at improving emotional states (Hayes et al., 2016). The previous literature is consistent with the role of acceptance in the suffering of FM patients (McCracken and Velleman, 2010). It has been proven that chronic pain patients with high levels of acceptance have a better state of mental health (Kanzler et al., 2019). The results found here may be due to the field of application of the technique. In recent years, the use of ACT in patients with FM has spread (Simister et al., 2018), but this therapy has been applied fundamentally to reduce the psychological symptoms associated with pain (i.e., anxiety or depression) but not in the reduction of pain itself (Graham et al., 2016).

The moderation analysis add that the variables of physical symptoms (pain, functional limitation, and physical fatigue) were significantly and negatively associated with acceptance, only in patients from the rural area. That is, in rural patients, the greater the acceptance, the less pain, functional limitation, and physical fatigue. However, in urban patients there is no association between these variables. As mentioned above, the sociodemographic characteristics of patients from rural areas compared to those from urban areas differ significantly. The rural environment favors continuing a similar lifestyle despite the disease. By not feeling substantial changes in their daily lives, it is possible that patients perceive less severity in physical symptoms. The characteristics of the environment and the lifestyle of rural women favor acceptance of the disease and the impact of it does not significantly affect their daily lives (Rahim-Williams et al., 2012). In this sense, our results indicate that the rural environment not only favors the acceptance of FM but also the positive effects of the latter on the symptoms associated with the disease.

Together, the results presented suggest that the area of residence plays a role in the severity and management of symptoms in patients with FM. This has important practical implications to consider. The findings make it clear that the characteristics of FM patients in rural and urban settings differ so much that studies should not generalize the results in this population. It is considered necessary to develop specific treatment programs taking into account the characteristics of the patients depending on the demographic area. Specifically, it is recommended that interventions in patients in urban areas, due to the impact that the disease can have on their lifestyle, are focused on its acceptance. Instead, in rural areas, efforts should focus on increasing resources for specialized care for chronic pain.

While acknowledging the relevant practical implications of the present research, this study certainly has some limitations. First, the associations must be interpreted according to the observational nature of the design, as this is not an experimental study. Furthermore, since the present study focuses exclusively on women with FM, the generalizability of the findings to other populations of musculoskeletal and non-musculoskeletal pain cannot be guaranteed. As the literature indicates, important differences have been revealed between this population and other populations with chronic pain (Arnold et al., 2016). Therefore, researchers are encouraged to replicate these findings in different pain populations. Finally, it is important to mention the absence of studies conducted in FM that explore the differences between rural and urban patients makes it difficult to compare results. Given the important clinical repercussions that these studies can have on the health system, it would be advisable to carry out more research in this line.

## Data Availability Statement

The original contributions presented in the study are included in the article/supplementary material, further inquiries can be directed to the corresponding author.

## Ethics Statement

The study was approved by the Bioethics Committee of Rey Juan Carlos University (Reference PI17/00858; number 160520165916). The patients/participants provided their written informed consent to participate in this study.

## Author Contributions

PC and CP contributed equally to this article in terms of study conceptualization, design, and procurement of funding. SB and SP-C organized data collection. OL-R analyzed the data. DB contributed to the development of the assessment materials. All authors contributed to the writing of the manuscript.

## Conflict of Interest

The authors declare that the research was conducted in the absence of any commercial or financial relationships that could be construed as a potential conflict of interest.

## References

[B1] Arnold L. M. Clauw D. J. (2017). Challenges of implementing fibromyalgia treatment guidelines in current clinical practice. *Postgrad. Med.* 129 709–714. 28562155 10.1080/00325481.2017.1336417

[B2] Arnold L. M. Choy E. Clauw D. J. Goldenberg D. L. Harris R. E. Helfenstein M. (2016). Fibromyalgia and chronic pain syndromes: a white paper detailing current challenges in the field. *Clin. J. Pain* 32 737–746. 10.1097/AJP.0000000000000354 27022674 PMC4974061

[B3] Blazer D. (1985). Psychiatric disorders. *Arch. Gen. Psychiatry* 42:651. 10.1001/archpsyc.1985.01790300013002 4015306

[B4] Bogusz R. Humeniuk E. (2017). Psychosocial determinants of disease acceptance in selected mental disorders. *Ann. Agric. Environ. Med.* 24 644–647. 10.5604/12321966.1235164 29284241

[B5] Brooks S. K. Rimes K. A. Chalder T. (2011). The role of acceptance in chronic fatigue syndrome. *J. Psychosom. Res.* 71 411–415. 10.1016/j.jpsychores.2011.08.001 22118384

[B6] Calandre E. P. Rico-Villademoros F. Galán J. Molina-Barea R. Vilchez J. S. Rodriguez-Lopez C. M. (2014). Quetiapine extended-release (Seroquel-XR) versus amitriptyline monotherapy for treating patients with fibromyalgia: a 16-week, randomized, flexible-dose, open-label trial. *Psychopharmacology (Berl)* 231 2525–2531. 10.1007/s00213-013-3422-0 24398824

[B7] Calkins M. P. (2018). “Memory care and Alzheimer’s units,” in *Environmental Psychology and Human Well-Being*, ed. Devlin A. S. (Amsterdam: Elsevier), 365–386. 10.1016/B978-0-12-811481-0.00014-7

[B8] Campos R. P. Vázquez M. I. R. (2012). Health-related quality of life in women with fibromyalgia: clinical and psychological factors associated. *Clin. Rheumatol.* 31 347–355. 10.1007/s10067-011-1870-7 21979445

[B9] Cheng C. Yang C. Inder K. Wai-Chi Chan S. (2020). Urban–rural differences in mental health among Chinese patients with multiple chronic conditions. *Int. J. Ment. Health Nurs.* 29 224–234. 10.1111/inm.12666 31609539

[B10] Chiu H.-C. Chen C.-M. Huang C.-J. Mau L.-W. (2005). Depressive symptoms, chronic medical conditions and functional status: a comparison of urban and rural elders in Taiwan. *Int. J. Geriatr. Psychiatry* 20 635–644. 10.1002/gps.1292 16021655

[B11] Ciapetti A. Salaffi F. Franchignoni F. Giordano A. Sarzi Puttini P. Ottonello M. (2013). SAT0387 psychometric characteristics of the italian version of the revised fibromyalgia impact questionnaire using classical test theory and rasch analysis. *Ann. Rheum. Dis.* 72 3–714. 10.1136/annrheumdis-2013-eular.2112 23806265

[B12] Cipora E. Konieczny M. Sobieszczañski J. (2018). Acceptance of illness by women with breast cancer. *Ann. Agric. Environ. Med.* 25 167–171. 10.26444/aaem/75876 29575856

[B13] Cleeland C. S. Ryan K. M. (1994). Pain assessment: global use of the Brief pain inventory. *Ann. Acad. Med. Singapore* 23 129–138. 8080219

[B14] Cohen J. (1988). *Statistical Power Analysis for the Behavioral Sciences.* Hillsdale, NJ: Lawrence Erlbaum Associates.

[B15] Dorado K. Schreiber K. L. Koulouris A. Edwards R. R. Napadow V. Lazaridou A. (2018). Interactive effects of pain catastrophizing and mindfulness on pain intensity in women with fibromyalgia. *Heal. Psychol. Open* 5:205510291880740. 10.1177/2055102918807406 30364853 PMC6198401

[B16] Fitzcharles M.-A. Rampakakis E. Ste-Marie P. A. Sampalis J. S. Shir Y. (2014). The association of socioeconomic status and symptom severity in persons with fibromyalgia. *J. Rheumatol.* 41 1398–1404. 10.3899/jrheum.131515 24931954

[B17] García D. Á Nicolás I. M. Hernández P. J. S. (2016). Clinical approach to fibromyalgia: synthesis of evidence-based recommendations, a systematic review. *Clin. Rheumatol.* 12 65–71. 10.1016/j.reuma.2015.06.001 26481494

[B18] Goh J. M. Gao G. Agarwal R. (2016). The creation of social value: can an online health community reduce rural–urban health disparities? *MIS Q.* 40 247–263.

[B19] Graham C. D. Gouick J. Krahé C. Gillanders D. (2016). A systematic review of the use of acceptance and commitment therapy (ACT) in chronic disease and long-term conditions. *Clin. Psychol. Rev.* 46 46–58. 10.1016/j.cpr.2016.04.009 27176925

[B20] Häuser W. Ablin J. Fitzcharles M.-A. Littlejohn G. Luciano J. V. Usui C. (2015). Fibromyalgia. *Nat. Rev. Dis. Prim.* 1:15022. 27189527 10.1038/nrdp.2015.22

[B21] Hayes A. F. (2017). *Introduction to Mediation, Moderation, and Conditional Process Analysis Second Edition A Regression-Based Approach.* New York, NY: The Guilford Press.

[B22] Hayes S. C. Luoma J. Bond F. W. Masuda A. Lillis J. (2016). Acceptance and commitment therapy: model, processes, and outcomes. *Act. Context Canonical Pap.* 249–279. 10.4324/9781315745138 16300724

[B23] Herrero M. J. Blanch J. Peri J. M. De Pablo J. Pintor L. Bulbena A. (2003). A validation study of the hospital anxiety and depression scale (HADS) in a Spanish population. *Gen. Hosp. Psychiatry* 25 277–283. 10.1016/S0163-8343(03)00043-4 12850660

[B24] Higuchi D. (2020). Adaptive and maladaptive coping strategies in older adults with chronic pain after lumbar surgery. *Int. J. Rehabil. Res.* 43 116–122. 10.1097/MRR.0000000000000389 31842023

[B25] Hogg M. N. Gibson S. Helou A. DeGabriele J. Farrell M. J. (2012). Waiting in pain: a systematic investigation into the provision of persistent pain services in Australia. *Med. J. Aust.* 196 386–390. 10.5694/mja12.10140 22471539

[B26] Huygens M. W. Vermeulen J. Swinkels I. C. Friele R. D. Van Schayck O. C. De Witte L. P. (2016). Expectations and needs of patients with a chronic disease toward self-management and eHealth for self-management purposes. *BMC Health Serv. Res.* 16:232. 10.1186/s12913-016-1484-5 27391471 PMC4938915

[B27] IBM Corp (2013). *IBM SPSS Statistics for Windows, Version 22.0.* Armonk, NY: IBM Corp.

[B28] Jao Y.-L. Liu W. Chaudhury H. Parajuli J. Holmes S. Galik E. (2020). Function-Focused person–environment fit for long-term care residents with dementia: impact on apathy. *Gerontologist* 61 413–424. 10.1093/geront/gnaa111 32833010

[B29] Jensen M. P. Turner L. R. Turner J. A. Romano J. M. (1996). The use of multiple-item scales for pain intensity measurement in chronic pain patients. *Pain* 67 35–40. 10.1016/0304-3959(96)03078-3 8895229

[B30] Jiao J. Vincent A. Cha S. S. Luedtke C. A. Oh T. H. (2014). Relation of age with symptom severity and quality of life in patients with fibromyalgia. *Mayo Clin. Proc.* 89 199–206. 10.1016/j.mayocp.2013.09.021 24485133

[B31] Kanzler K. E. Pugh J. A. McGeary D. D. Hale W. J. Mathias C. W. Kilpela L. S. (2019). Mitigating the effect of pain severity on activity and disability in patients with chronic pain: the crucial context of acceptance. *Pain Med.* 20 1509–1518. 10.1093/pm/pny197 30590737 PMC6686120

[B32] Kratz A. L. Davis M. C. Zautra A. J. (2007). Pain acceptance moderates the relation between pain and negative affect in female osteoarthritis and fibromyalgia patients. *Ann. Behav. Med.* 33 291–301. 10.1007/BF02879911 17600456 PMC2593934

[B33] Kress H.-G. Aldington D. Alon E. Coaccioli S. Collett B. Coluzzi F. (2015). A holistic approach to chronic pain management that involves all stakeholders: change is needed. *Curr. Med. Res. Opin.* 31 1743–1754. 10.1185/03007995.2015.1072088 26172982

[B34] Lami M. J. Martínez M. P. Miró E. Sánchez A. I. Guzmán M. A. (2018). Catastrophizing, acceptance, and coping as mediators between pain and emotional distress and disability in fibromyalgia. *J. Clin. Psychol. Med. Settings* 25 80–92. 10.1007/s10880-018-9543-1 29450798

[B35] Lawson K. (2017). Emerging pharmacological strategies for the treatment of fibromyalgia. *World J. Pharmacol.* 6 1–10. 10.5497/wjp.v6.i1.1

[B36] Lawton M. P. (1977). “The impact of the environment on aging and behaviour,” in *New Dimensions in Environmental Desi gn Research*, eds Birren J. Schaie W. (New York, NY: Van Nostrand Reinhold), 276–301.

[B37] Lawton M. P. (1986). *Environment and Aging*, 2nd Edn. Albany, NY: Center for the Study of Aging.

[B38] LeRoux H. Fisher J. E. (2006). “Strategies for enhancing medication adherence in the elderly,” in *Promoting Treatment Adherence: A Practical Handbook for Health Care Providers*, eds O’Donohue W. T. Levensk E. R. (Thousand Oaks, CA: SAGE Publications, Inc.), 353–362. 10.4135/9781452225975.n24

[B39] Li L. W. Liu J. Xu H. Zhang Z. (2016). Understanding rural–urban differences in depressive symptoms among older adults in China. *J. Aging Health* 28 341–362. 10.1177/0898264315591003 26100620 PMC4893784

[B40] Marques A. P. Santo A. de S. do E. Berssaneti A. A. Matsutani L. A. (2017). Prevalence of fibromyalgia: literature review update. *Rev. Bras. Reumatol. (English Ed.)* 57 356–363. 10.1016/j.rbre.2017.01.005 28743363

[B41] Mayangsari E. D. Poerwandari E. K. Christia M. (2019). “Pain management, coping with stress, and quality of life for women with fibromyalgia: a qualitative case study,” in *Proceedings of the 2nd International Conference on Intervention and Applied Psychology (ICIAP 2018)*, (Paris: Atlantis Press). 10.2991/iciap-18.2019.24

[B42] McCracken L. M. Velleman S. C. (2010). Psychological flexibility in adults with chronic pain: a study of acceptance, mindfulness, and values-based action in primary care. *Pain* 148 141–147. 10.1016/j.pain.2009.10.034 19945795

[B43] McCracken L. M. Vowles K. E. Eccleston C. (2004). Acceptance of chronic pain: component analysis and a revised assessment method. *Pain* 107 159–166. 10.1016/j.pain.2003.10.012 14715402

[B44] Ministerio de Transportes, Movilidad y Agenda Urbana, and Direccion General de Vivienda y Suelo (2020). *Áreas Urbanas en España 2019.* Madrid: Centro de Publicaciones del Ministerio de Transportes, Movilidad y Agenda Urbana.

[B45] Moore K. D. VanHaitsma K. Curyto K. Saperstein A. (2003). A pragmatic environmental psychology: a metatheoretical inquiry into the work of M. Powell Lawton. *J. Environ. Psychol.* 23 471–482. 10.1016/S0272-4944(02)00116-0

[B46] Munguía-Izquierdo D. Segura-Jimenez V. Camiletti-Moiron D. Pulido-Martos M. Álvarez-Gallardo I. C. Romero A. (2012). Multidimensional fatigue inventory: Spanish adaptation and psychometric properties for fibromyalgia patients. The Al-andalus study. *Clin. Exp. Rheumatol.* 30 94–102. 23261007

[B47] Murawski L. Church R. L. (2009). Improving accessibility to rural health services: the maximal covering network improvement problem. *Socioecon. Plann. Sci.* 43 102–110. 10.1016/j.seps.2008.02.012

[B48] Perry T. E. Andersen T. C. Kaplan D. B. (2014). Relocation remembered: perspectives on senior transitions in the living environment. *Gerontologist* 54 75–81. 10.1093/geront/gnt070 23840021 PMC4118138

[B49] Pidolle M.-A. El Hage W. (2020). Fibromyalgia ans psycho-traumatic stress: psychotropic drugs and psychotherapies. *Rev. Prat.* 70 1011–1016. 33739764

[B50] Rahim-Williams B. Riley J. L. Williams A. K. K. Fillingim R. B. (2012). A quantitative review of ethnic group differences in experimental pain response: do biology, psychology, and culture matter? *Pain Med.* 13 522–540. 10.1111/j.1526-4637.2012.01336.x 22390201 PMC3349436

[B51] Rodero B. Casanueva B. Luciano J. V. Gili M. Serrano-Blanco A. García-Campayo J. (2011). Relationship between behavioural coping strategies and acceptance in patients with fibromyalgia syndrome: elucidating targets of interventions. *BMC Musculoskelet. Disord.* 12:143. 10.1186/1471-2474-12-143 21714918 PMC3150336

[B52] Rodero B. García-Campayo J. Casanueva B. del Hoyo Y. L. Serrano-Blanco A. Luciano J. V. (2010). Veaselaircdhation of the Spanish version of the Chronic Pain Acceptance Questionnaire (CPAQ) for the assessment of acceptance in fibromyalgia. *Health Qual. Life Outcomes* 8:37. 20385016 10.1186/1477-7525-8-37 PMC2876109

[B53] Rousseau J. Potvin L. Dutil E. Falta P. (2002). Model of competence: a conceptual framework for understanding the person-environment interaction for persons with motor disabilities. *Occup. Ther. Heal. Care* 16 15–36. 10.1080/J003v16n01_02 23952060

[B54] Salgueiro M. García-Leiva J. M. Ballesteros J. Hidalgo J. Molina R. Calandre E. P. (2013). Validation of a Spanish version of the Revised Fibromyalgia Impact Questionnaire (FIQR). *Health Qual. Life Outcomes* 11:132. 10.1186/1477-7525-11-132 23915386 PMC3770447

[B55] Salinas J. J. Al Snih S. Markides K. Ray L. A. Angel R. J. (2010). The rural-urban divide: health services utilization among older Mexicans in Mexico. *J. Rural Heal.* 26 333–341. 10.1111/j.1748-0361.2010.00297.x 21029168 PMC2967463

[B56] Sanz-Baños Y. Pastor M. -Á Velasco L. López-Roig S. Peñacoba C. Lledo A. (2016). To walk or not to walk: insights from a qualitative description study with women suffering from fibromyalgia. *Rheumatol. Int.* 36 1135–1143. 10.1007/s00296-016-3459-6 26979604

[B57] Simister H. D. Tkachuk G. A. Shay B. L. Vincent N. Pear J. J. Skrabek R. Q. (2018). Randomized controlled trial of online acceptance and commitment therapy for fibromyalgia. *J. Pain* 19 741–753. 10.1016/j.jpain.2018.02.004 29481976

[B58] Song Y. Tan Y. Song Y. Wu P. Cheng J. C. Kim M. J. (2018). Spatial and temporal variations of spatial population accessibility to public hospitals: a case study of rural–urban comparison. *GIScience Rem. Sens.* 55 718–744. 10.1080/15481603.2018.1446713

[B59] St John P. D. Blandford A. A. Strain L. A. (2006). Depressive symptoms among older adults in urban and rural areas. *Int. J. Geriatr. Psychiatry* 21 1175–1180. 10.1002/gps.1637 16988957

[B60] Suso-Ribera C. García-Palacios A. Botella C. Ribera-Canudas M. V. (2017). Pain catastrophizing and its relationship with health outcomes: does pain intensity matter? *Pain Res. Manag.* 2017:9762864. 10.1155/2017/9762864 28348506 PMC5350380

[B61] Tangen S. F. Helvik A.-S. Eide H. Fors E. A. (2020). Pain acceptance and its impact on function and symptoms in fibromyalgia. *Scand. J. Pain* 20 727–736. 10.1515/sjpain-2020-0049 32759409

[B62] Thompson M. McCracken L. M. (2011). Acceptance and related processes in adjustment to chronic pain. *Curr. Pain Headache Rep.* 15 144–151. 10.1007/s11916-010-0170-2 21222244

[B63] Trainor H. Baranoff J. Henke M. Winefield H. (2019). Functioning with fibromyalgia: the role of psychological flexibility and general psychological acceptance. *Aust. Psychol.* 54 214–224. 10.1111/ap.12363

[B64] Tripp D. A. VanDenKerkhof E. G. McAlister M. (2006). Prevalence and determinants of pain and pain-related disability in urban and rural settings in Southeastern Ontario. *Pain Res. Manag.* 11 225–233. 10.1155/2006/720895 17149455 PMC2673139

[B65] Van Liew C. Leon G. Neese M. Cronan T. A. (2019). You get used to it, or do you: symptom length predicts less fibromyalgia physical impairment, but only for those with above-average self-efficacy. *Psychol. Health Med.* 24 207–220. 10.1080/13548506.2018.1524152 30270643 PMC6338553

[B66] Von Elm E. Altman D. G. Egger M. Pocock S. J. Gøtzsche P. C. Vandenbroucke J. P. (2008). The strengthening the reporting of observational studies in epidemiology (STROBE) statement: guidelines for reporting observational studies. *J. Clin. Epidemiol.* 61 344–349. 18313558 10.1016/j.jclinepi.2007.11.008

[B67] Walitt B. Nahin R. L. Katz R. S. Bergman M. J. Wolfe F. (2015). The prevalence and characteristics of fibromyalgia in the 2012 national health interview survey. *PLoS One* 10:e0138024. 10.1371/journal.pone.0138024 26379048 PMC4575027

[B68] Wang J. L. (2004). Rural–urban differences in the prevalence of major depression and associated impairment. *Soc. Psychiatry Psychiatr. Epidemiol.* 39 19–25. 15022042 10.1007/s00127-004-0698-8

[B69] Wang S. Kou C. Liu Y. Li B. Tao Y. D’Arcy C. (2015). Rural–Urban differences in the prevalence of chronic disease in Northeast China. *Asia Pac. J. Public Heal.* 27 394–406. 10.1177/1010539514551200 25246500

[B70] Westland J. C. (2010). Lower bounds on sample size in structural equation modeling. Electronic commerce research and applications. *Electron. Commer. Res. Appl.* 9 476–487.

[B71] Wolfe F. Clauw D. J. Fitzcharles M. A. Goldenberg D. L. Katz R. S. Mease P. (2010). The American college of rheumatology preliminary diagnostic criteria for fibromyalgia and measurement of symptom severity. *Arthritis Care Res.* 62 600–610. 10.1002/acr.20140 20461783

[B72] Wolfe F. Smythe H. A. Yunus M. B. Bennett R. M. Bombardier C. Goldenberg D. L. (1990). The American college of rheumatology 1990 criteria for the classification of fibromyalgia. report of the multicenter criteria committee. *Arthritis Rheum.* 33 160–172. 10.1016/j.pain.2008.02.009 2306288

[B73] Wolfe F. Walitt B. T. Katz R. S. Häuser W. (2014). Symptoms, the nature of fibromyalgia, and diagnostic and statistical manual 5 (DSM-5) defined mental illness in patients with rheumatoid arthritis and fibromyalgia. *PLoS One* 9:e88740. 10.1371/journal.pone.0088740 24551146 PMC3925165

[B74] Yu L. Norton S. Almarzooqi S. McCracken L. M. (2017). Preliminary investigation of self-as-context in people with fibromyalgia. *Br. J. Pain* 11 134–143. 10.1177/2049463717708962 28785409 PMC5521349

[B75] Zimmer Z. Wen M. Kaneda T. (2010). A multi-level analysis of urban/rural and socioeconomic differences in functional health status transition among older Chinese. *Soc. Sci. Med.* 71 559–567. 10.1016/j.socscimed.2010.03.048 20621749 PMC2904335

